# Targeted ferroptotic potency of ferrous oxide nanoparticles-diethyldithiocarbamate nanocomplex on the metastatic liver cancer

**DOI:** 10.3389/fphar.2022.1089667

**Published:** 2023-01-04

**Authors:** Marwa M. Abu-Serie

**Affiliations:** Medical Biotechnology Department, Genetic Engineering and Biotechnology Research Institute, City of Scientific Research and Technological Applications (SRTA-City), Alexandria, Egypt

**Keywords:** metastatic liver cancer, selective ferroptosis, ferrous oxide NPs-diethyldithiocarbamate nanocomplex, blocking antioxidant (anti-ferroptotic) mediators, cancer stem cell genes

## Abstract

Existing treatments are frequently ineffective in combating liver cancer (LC) due to its rapid growth, high metastatic potential, and chemoresistance. Thus, inducing ferroptosis, a new non-apoptotic regulated cell death-dependent massive iron overload-mediated lipid peroxidation, is an alternative effective approach for treating LC. The efficient trigger of ferroptosis requires blocking cellular antioxidant (anti-ferroptosis) response and selectivity to avoid harming other healthy tissues. In this study, green chemically synthesized ferrous oxide nanoparticles (F(II) NPs) were used for enhancing selective iron accumulation in tumor tissue, while diethyldithiocarbamate (DE) was for inhibiting the antioxidant system (glutathione and aldehyde dehydrogenase (ALDH) 2) which protects the tumor from damage-dependent lipid peroxides. Thus, F(II) NPs were used with DE as a nanocomplex (DF(II) NPs), whose anti-LC activity was compared to that of the typical complex, DF(II). In HepG2 cells and a chemically induced metastatic LC animal model, DF(II) NPs outperformed DF(II) in eradicating metastatic LC cells, as evidenced by flow cytometry, histological and immunohistochemical analyses, and α-fetoprotein depletion. The superior therapeutic potency-dependent ferroptotic activity of DF(II) NPs, attributed to their higher selective accumulation (∼77%) than DF(II) in tumor tissues (liver and lung), resulted in a strong elevation of cellular lipid peroxidation with extreme suppression of nuclear related factor 2 (Nrf2) transcriptional activity, glutathione (GSH), glutathione peroxidase 4, and ALDH2. Subsequently, a severe inhibition in the expression of oncogenes and metastatic cancer stem cell genes was recorded in DF(II) NPs-treated LC animal group. In contrast to DF(II), DF(II) NPs were able to normalize liver functions and did not show any variations in hematological and histological parameters in the blood and tissues of DF(II) NPs-treated normal mouse group. These findings validate the potency and safety of DF(II) nanocomplex as a promising nanodrug for combating metastatic LC.

## 1 Introduction

Liver cancer (LC) is a lethal malignancy that affects people all over the world. Because of its rapid proliferation, and migration as well as chemoresistance, its progression is difficult to be controlled with existing treatment options, particularly at the advanced stage (metastasis), resulting in a low survival rate (Li et al., 2022; Zhao et al., 2022). Consequently, finding an alternative remedies becomes a current concern. One of the effective therapeutic strategies is inducing ferroptosis, a new non-apoptotic form of regulated cell death which is characterized by iron accumulation-mediated lipid peroxidation propagation (Bekric et al., 2022; Li et al., 2022). It is important to note that unselective induction of ferroptosis can also damage other healthy hepatic cells and tissues (Bekric et al., 2022). Therefore, a nanoformulation of ferroptotic inducers is critical for improving safety by preventing unspecific side effects and enhancing anti-cancer efficacy.

In this study, green chemically synthesized ferrous oxide nanoparticles (F(II) NPs) were used as a ferroptosis inducer. These NPs mediate iron accumulation inside tumor tissues based on the enhanced permeability and retention effect (Wilhelm et al., 2016). Excess iron mediates, *via* the Fenton reaction, the uncontrolled generation of hydroxyl radicals initiating lipid peroxidation chains that produce reactive aldehydes, causing damage to cellular components (Wan et al., 2019). In response to ferroptosis, cancer cells upregulate and activate nuclear related factor 2 (Nrf2), a master regulator of antioxidant response that mediates transcription and expression of enzymatic and non-enzymatic antioxidants (including, glutathione (GSH) and glutathione peroxidase (GPX)4, respectively). It was found that GPX4 is overexpressed in advanced malignant stage in LC patients (Bekric et al., 2022). This glutathione system is critical for protecting cancer cells against ferroptosis-dependent death *via* the detoxification activity of GPX4 for reducing lipid peroxides into their alcohols using GSH as a cofactor. The latter itself can also reduce lipid peroxides (Yang et al., 2020; Bekric et al., 2022). Thus, it is necessary to halt the antioxidant-mediated anti-ferroptotic response of cancer cells by combining F(II) NPs with a prooxidant agent (such as diethyldithiocarbamate, a metabolite of FDA-approved alcoholism remedy). Diethyldithiocarbamate (DE) has high affinity to F(II) NPs and is a powerful inhibitor of aldehyde dehydrogenase (ALDH)2 that metabolizes lipid peroxidation-derived aldehydes to protect cancer stem cell functions (Wang et al., 2020; Abu-Serie & Abdelfattah, 2022). Hepatic cancer stem cells (CSCs) are primarily responsible for the initiation, chemoresistance, and metastasis of LC (Liu et al., 2020). It was found that ALDH2 overexpression is related to the aggressive progression of LC (Wang et al., 2020) *via* maintaining stemness and invasion features of hepatic CSCs (Zhang & Fu, 2021). Furthermore, DE caused GSH depletion thus mediating an apoptosis-dependent oxidative stress and exacerbated ferroptosis (Abu-Serie & Abdelfattah, 2022). A recent study illustrated that nanocomplex of DE with F(II) NPs (DF(II) NPs) exhibited a higher therapeutic potential than DE-ferric oxide NPs for eradicating metastatic breast tumor using orthotopic animal model (Abu-Serie & Abdelfattah, 2022).

Herein, the anticancer activity of this nanocomplex (DF(II) NPs) was investigated, compared to DE complex with ferrous oxide (DF(II)), and their individual components (DE, F(II) NPs, and F(II)) against HepG2 cells. Then, therapeutic potentials of both complexes (DF(II) NPs and DF(II)) were evaluated using chemically induced metastatic liver tumor animal model through assessing the elevation in reactive oxygen species (ROS) and lipid peroxidation and the suppression of the anti-ferroptotic parameters (Nrf2, GSH, GPX4, and ALDH2). The impact of DE-F(II) NPs-mediated ferroptosis on CSCs genes, including stemness, chemoresistance, and metastasis was also investigated.

## 2 Materials and methods

### 2.1 Materials

Iron nitrate, ferrous oxide, MTT, nuclear fluorescence dyes, *p*-dimethylaminobenzene (DMAB), phenobarbital, 2′-7′-Dichlorodihydrofluorescein diacetate (DCFD), thiobarbituric acid (TBA), Ellman’s reagent, GSH, glutathione reductase, cumene hydroperoxide, NADPH, and NAD were obtained from Sigma-Aldrich (United States). DE was from Acros Organics (United States). RPMI 1640 medium and fetal bovine serum (FBS) were acquired from GIBCO (United States). Primary antibodies of ki-67 and N-cadherin, RNA extraction kit, primers, and one-step qPCR SYBR green kit were purchased from Thermo Fisher Scientific (United States). α-Fetoprotein (AFP) electrochemiluminescence kit and Nrf2 transcription factor kit were purchased from Roche Diagnostics (United States) and Abcam (UK), respectively. Liver function kits were acquired from Spectrum Diagnostics company (Egypt).

### 2.2 Preparation of DF(II) complexes

Ferrous oxide NPs were prepared by the addition of vitamin C (20%) to a mixture of 160 mM Fe(NO_3_)_3_.9H_2_O and 1N NaOH and centrifugation as described previously (Abu-Serie & Abdelfattah, 2022). The characterized F(II) NPs (0.02 mg/ml) were mixed with DE (0.2 mg/ml) forming a soluble nanocomplex of DF(II) NPs whose morphology was investigated using transmission and scanning electron microscopes (JEOL, JEM-1230, Japan and JEOL JSM5300, Japan, respectively) and diameter was measured by ImageJ software. The traditional complex of ferrous oxide and DE was prepared in the same ratio (0.1:1).

### 2.3 *In vitro* investigation of anti-liver cancer activity

#### 2.3.1 MTT assay

Briefly, HepG2 cells which were cultured in 10% FBS-supplemented RPMI 1640 medium, seeded (5 × 10^3^) in 96-well culture plate. After 24 h in CO_2_ incubator (37 °C), cells were incubated for 72 h with serial concentrations of DF(II) NPs, DF(II), DE, F(II)NPs, and FeO. According to Mosmann (1983), MTT assay was used to assess the percentage of growth inhibition in the treated cells relative to the untreated cells and then the dose at which 50% growth inhibition (IC_50_) was estimated using GraphPad Prism 9. Additionally, morphological alterations in chelating complexes (the most active compounds)-treated HepG2 cells were recorded using phase contrast inverted microscope (Olympus, Japan).

#### 2.3.2 Lipid peroxidation assay (ferroptosis marker)

Briefly, HepG2 cells were treated with the above-mentioned compounds (at the lowest IC_50_ values “3.4 μg/ml”). After 72 h, the elevation in cellular content of lipid peroxidation was determined in the supernatant, relative to the untreated cells, using thiobarbituric acid reactive substance method (Girotti et al., 1991).

#### 2.3.3 Fluorescence nuclear staining for investigation of cell death

After 72 h treatment of HepG2 with 3.4 μg/ml of DF complexes (DF(II) NPs and DF(II)). Cell death can be observed after staining the untreated and treated cells with acridine orange/ethidium bromide (AO/EB) using fluorescence microscope (Olympus, Japan). For accurate quantification, cells were stained with fluorescein isothiocyanate (FITC)-labelled annexin V/propidium iodide (PI) and analyzed at FITC signal detector (FL1) and phycoerythrin emission signal detector (FL2) using flow cytometry (Partec, Netherlands) with FloMax software.

#### 2.3.4 Wound healing (migration) assay

The anti-migration activity of DF complexes was evaluated by 24 h incubation with scratched monolayer HepG2. The wound closure area was recorded, by a digital camera-phase contrast inverted microscope, at 0 h and 24 h of treatment and then analyzed using ImageJ NIH software.

### 2.4 *In vivo* investigation of anti-liver cancer activity

About 75 male Swiss albino mice (20–25 g) were split into two main groups, healthy group (N, n = 36) and liver cancer group (LC, n = 39). LC group was supplied with 165 mg DMAB/kg body weight (b.w) and oral administered with 0.05% phenobarbital (1.2 mg/kg b. w) daily for 6 weeks **(** Pathak & Khuda-Bukhsh, 2007 **)** for inducing LC. After 6 weeks, the metastatic LC incidence was clarified by hematoxylin and eosin (H&E) staining of liver and lung tissues (Figure 2D) from three sacrificed LC mice. Then these two groups (N and LC) were blindly divided into three sub-groups, including untreated, DF(II) NPs, and DF(II). The two treated groups of each main group (i.e., N-DF(II) NPs, N-DF(II), LC-DF(II) NPs, and LC-DF(II)) were intraperitoneally injected with 2 mg DF complexes/kg b. w for 3 weeks (three times/week). At the eighth week, six mice from each group were decapitated under isoflurane anesthesia, and then liver and lung organs were gathered for the analysis of redox indicators (ROS, Nrf2, GSH, GPX4, and ALDH2). At the end of this experiment (9th week), all mouse groups were sacrificed after isoflurane-anaesthetizing then blood and tissues were harvested. For hematological and AFP tests, blood samples were drawn in EDTA tubes. Liver, lung, and spleen tissues were weighted in relation to the measured body weight. Small parts of tissues were 10% formalin-fixed for histological and immunohistochemical assessments, whilst the remaining parts were kept (−80°C) for biochemical and molecular investigations.

#### 2.4.1 Histological and immunohistochemical examinations of tumor tissues and quantification of α-fetoprotein level

Typical procedures were used to prepare the tissue slides for the histological investigation of liver, lung, and spleen by H&E staining and immunohistochemical assessments (ki-67 immunostaining tumor tissues “liver and lung” as well as N-cadherin immunostaining liver). CellSens and ImageJ were used to analyze the immunostaining images.

The level of AFP (a basic liver tumor marker) in the blood was quantified using the AFP-chemiluminescent immunoassay kit.

#### 2.4.2 Atomic absorption quantification for biodistribution of DF(II) NPs and DF(II)

In a separate animal study, LC-bearing mice (b.w ∼ 20 g, n = 15) were divided into three groups (untreated LC, LC-DF(II) NPs, and LC-DF). The two treated LC groups were received intraperitoneally 0.04 mg of DF complexes per day for 1 week. After that, mice were sacrificed and tissues (hepatic nodules, lung, spleen, brain, heart, and kidney) were harvested. Using graphite atomic absorption spectrometry (Analytik Jena AG, Germany), the tissue level of iron which is equivalent to the accumulative tissue uptake of DF complexes, was quantified after normalization by subtracting from the corresponding iron tissue level of the untreated LC group.

#### 2.4.3 Quantification of redox parameters in tumor tissues

The following redox parameters were assessed in liver and lung tissue homogenates of the treated LC groups, relative to the untreated groups, after centrifugation at 3,000 *g* for 15 min at 4 °C. The fold increment in ROS and lipid peroxidation was determined using DCFD (Hempel et al., 1999) and TBA reactive substance (Ohkawa et al., 1979) methods, respectively. Furthermore, the relative fold decrement in antioxidant parameters (Nrf2 transcription activation and GSH content) were measured in these tumor tissue homogenates according to the instructions of Nrf2 transcription factor assay kit and Ellman’s assay (Ellman, 1959), respectively.

Briefly, following the tissue homogenization in 0.1 M Tris-HCl pH 7.4 (0.25 M sucrose, 5 mM β-mercaptoethanol, and protease inhibitor cocktail) and centrifugation, GPX4 activity was measured using coupled enzyme assay (Roveri et al., 1994). To begin reaction, tissue samples were added to the mixture of 0.14 U glutathione reductase, 0.2 mM NADPH, 3 mM GSH, 0.1 mM cumene hydroperoxide (substrate) and then oxidation of NADPH was recorded, per min, at 340 nm (Kernstock & Girotti, 2008). Regarding ALDH2 activity, in brief, the tissue homogenate supernatants (at 100,000 xg for 60 min at 4°C) were mixed with 1% triton and re-centrifuged, the obtained supernatants were then incubated with 50 mM sodium pyrophosphate buffer pH 9.5, 2.5 mM NAD^+^(coenzyme) and 10 μM malondialdehyde (substrate). ALDH2 activity was estimated as μmole NADH generated/min/mg protein (Josan et al., 2013). All of the aforementioned assays were normalized using the tissue protein content which was quantified using Bradford (1976) assay (Bradford, 1976).

#### 2.4.4 QPCR assessment for molecular tumor markers

Total RNA was isolated from the tumor tissues (liver and lung) of LC (untreated and treated) groups using the kit’s standard protocol. Following the quantification of RNA, one-step SYBR green master mix (qPCR kit) with certain primers (Supplementary Table S1) had been used to assess the relative variations in the gene expression using an equation of 2^−ΔΔCt^. These genes included Bcl-2-associated X protein (BAX), cyclin-dependent kinase inhibitor 1 (p21), cyclin D, telomerase reverse transcriptase (TERT), vascular endothelial growth factor (VEGF), matrix metalloprotease (MMP)9, ATP binding cassette subfamily G member 2 (ABCG2), CD90, NOTCH1, WNT1, sex-determining region Y-box 9 (Sox9), 4-octamer-binding transcription factor (OCT), and Nanog.

#### 2.4.5 Biochemical detection of liver functions and hematological parameters

The main liver function parameters were determined in liver homogenate supernatants (1 g liver homogenizing in 9 ml of 150 mM Tris-KCl buffer pH 7.4) after centrifugation at 3,000 *g* for 15 min at 4 °C (Gaskill et al., 2005). Using colorimetric kits, these parameters (alanine aminotransferase (ALT), aspartate aminotransferase (AST), and albumin) were assessed in both treated LC groups, relative to the untreated LC and healthy N group. A complete blood count (CBC) was also performed on all groups using a hematology analyzer (Mindray, China).

#### 2.4.6 Histological investigation of DF complexes impacts on the main tissues of the treated N groups

The safety of DF(II) complexes was examined using H&E staining of multiple tissues (liver, lung, spleen, brain, and kidney) of DF(II) NPs- and DF(II)-treated groups *versus* the untreated healthy N group.

### 2.5 Statistical analysis

Data are demonstrated as mean ± standard error of mean (SEM). GraphPad Prism 9.3.1. was used to analyze the obtained data using *t*-test and one-way analysis of variance (ANOVA) multiple comparison with Dunnett’s *post hoc* test. The statistical significance was considered at *p* ≤ 0.05*, ≤0.005**, and ≤0.001***.

## 3 Results and discussion

The use of nanoformulation of DF(II) to induce ferroptosis with blocking cellular antioxidant system is an alternative effective therapeutic approach for metastatic LC.

### 3.1 DF(II) NPs synthesis

**FIGURE 1:**
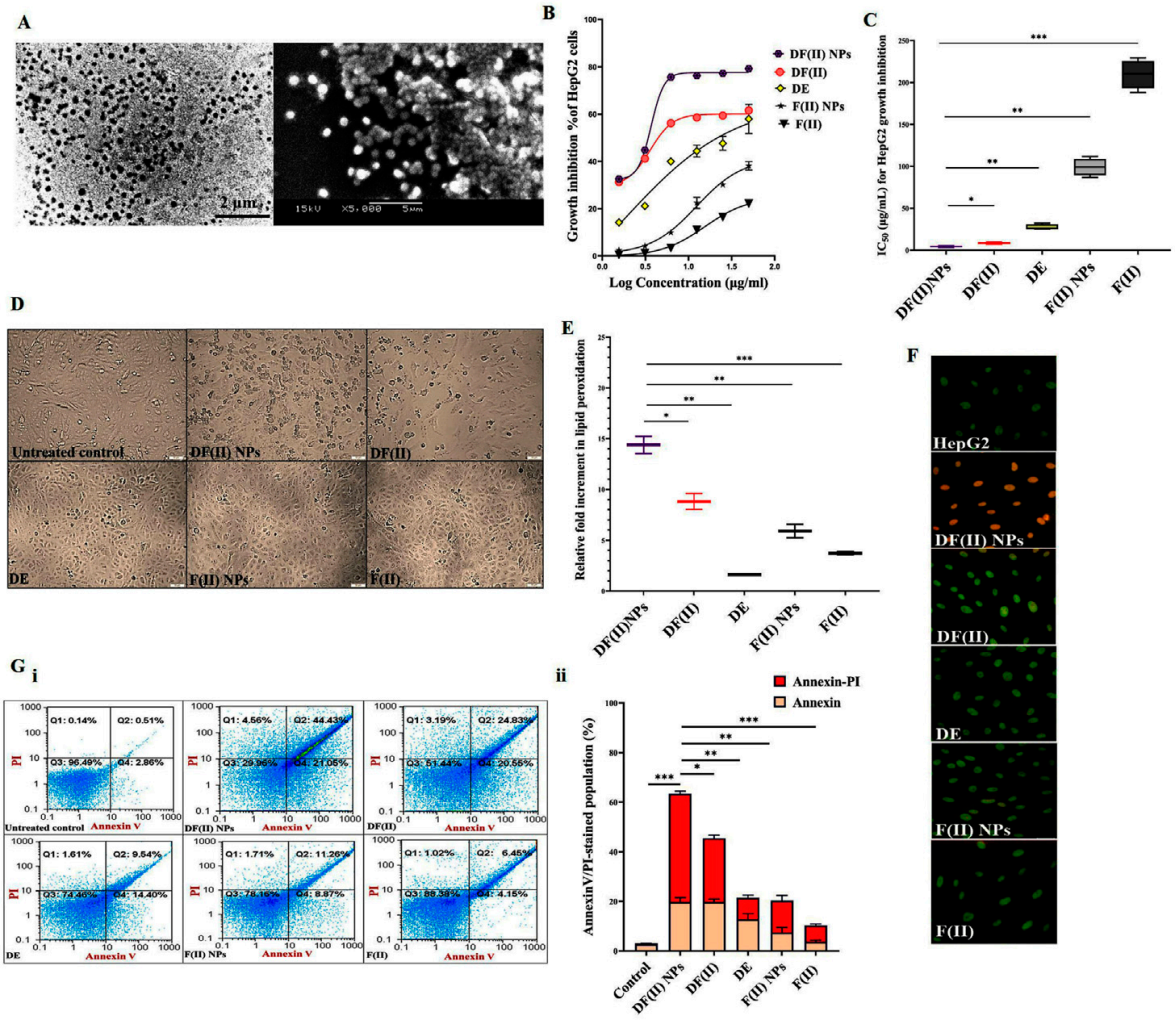
Morphology of ferrous oxide nanoparticles-diethyldithiocarbamate nanocomplex (DF(II) NPs) and its anticancer activity (*in vitro* study). **(A)** Micrograph of DF(II) NPs using transmission electron microscope (left image) and scanning electron microscope (right image) at magnification ×5000). MTT results of HepG2 growth inhibition as illustrated by **(B)** dose-response curve of both complexes, diethyldithiocarbamate (DE), ferrous oxide nanoparticles (FII NPs), and ferrous oxide (F(II)) and **(C)** their IC_50_ values with **(D)** morphological alterations of DF(II) NPs- and DF(II)-treated HepG2 cells using phase contrast microscope (Magnification ×100). **(E)** Relative fold increment in lipid peroxidation in the treated HepG2 cells. All values are demonstrated as mean ± SEM. DF(II) NPs-treated cells were compared to other treated cells and the differences were considered significant at *p* ≤ 0.05*, ≤0.005**, and ≤0.001*** **(F)** Fluorescence microscope after staining with acridine orange/ethidium bromide (Green, yellowish green and orange fluorescences indicating for viable, early death and late death stage cells, respectively). **(G)** Flow cytometry analysis of the treated HepG2 cells, after staining with annexin V/propidium iodide (annexin/PI) as showed by **(i)** dot plots (Q1, Q2, Q3, and Q4 refer to propidium iodide (PI)-stained cells (PI & annexin V) dual stained cells at the late death stage, negatively stained healthy cells, and annexin-stained early apoptotic cells, respectively) and **(ii)** the percentages of annexin- and annexin/PI-stained cell populations. All values are demonstrated as mean ± SEM. DF(II) NPs-treated cells were compared to other treated cells and the differences were considered significant at *p* ≤ 0.05*, ≤0.005**, and ≤0.001***.

The studied DF(II) NPs were prepared by using the chelating affinity of DE to F(II) NPs (1:0.1). These NPs were completely characterized their size (157.8 nm) with zeta potential (-41.25), morphology, oxidation state, and composition using zetasizer, scanning and transmission electron microscopes, X-ray diffraction, energy dispersive X-ray analysis, respectively, as showed in author’s recent study. Moreover, the formation of this nanocomplex was affirmed by fourier transformed infrared (IR) spectra (Abu-Serie & Abdelfattah, 2022). Figure 1A shows the semi-spherical morphology of as-prepared nanocomplex under transmission and scanning electron microscopes with the assessed diameter of 176.3 ± 4.13 nm.

### 3.2 *In vitro* anticancer activity of DF(II) NPs outperforming DF(II)

MTT results (Figures 1B,C) show that all tested compounds inhibited HepG2 growth in a dose-dependent manner and their growth inhibition potentials can be ranked as follows; DF(II) NPs > DF(II)> DE > F(II) NPs > F(II). In comparison to other compounds (IC_50_ ≥ 27 μg/ml), DF(II) NPs and DF(II) had the lowest IC_50_ values (4.27 ± 0.47 μg/ml and 8.31 ± 0.56 μg/ml, respectively). Accordingly, in the treated HepG2, DF(II) NPs exhibited a 2-fold higher anticancer potency with causing more dramatically alterations (Figure 1D) in cellular morphology than DF(II). The highest cytotoxicity of DF(II) complexes against LC cells attributes to efficient enhancement of F(II)-mediated ferroptosis, in the presence of DE as a prooxidant agent, that was confirmed by the highest elevation of lipid peroxidation (≥9 folds) in DF(II) complexes-treated HepG2 cells (Figure 1E). This current result is in line with author’s recent study, which illustrated that DE-treated MDA-MB 231 cells had a lower GSH level than F(II) NPs-treated cells. Subsequently, the highest fold increment of the cellular lipid peroxidation level, as indicator of ferroptosis, was recorded for DF(II) NPs compared to DE, F(II) NPs, and DE-ferric nitrate complex (Abu-Serie & Abdelfattah, 2022). Importantly, Figure 1E also shows that the relative elevation in cellular lipid peroxidation was significantly higher in DF(II) NPs-treated cells (14.39 ± 0.85 folds) than in DF(II)-treated cells (8.81 ± 0.789 folds). Furthermore, fluorescence staining with AO/EB illustrated orange fluorescence of DF(II) NPs-treated cells, and yellowish green fluorescence of DF(II)-treated cells indicating late stage cell death, and early stage cell death, respectively, compared to green fluorescence of the untreated healthy HepG2 nuclei. Meanwhile, DE-, F(II) NPs, and F(II)-treated cells exhibited light green fluorescence with the lowest number of yellowish green fluorescence nuclei (Figure 1F). AO/EB staining distinguishes between viable, early death, and late death based on the fact that AO intercalates into viable and non-viable cells’ DNA, giving them green fluorescence, but EB is only taken up by dying cells, giving them red fluorescence. Early apoptotic cells take up less EB than late apoptotic cells, whose membrane and DNA are completely damaged, resulting in yellowish green and orange-red fluorescence, respectively (García-Rodríguez et al., 2013).

**FIGURE 2:**
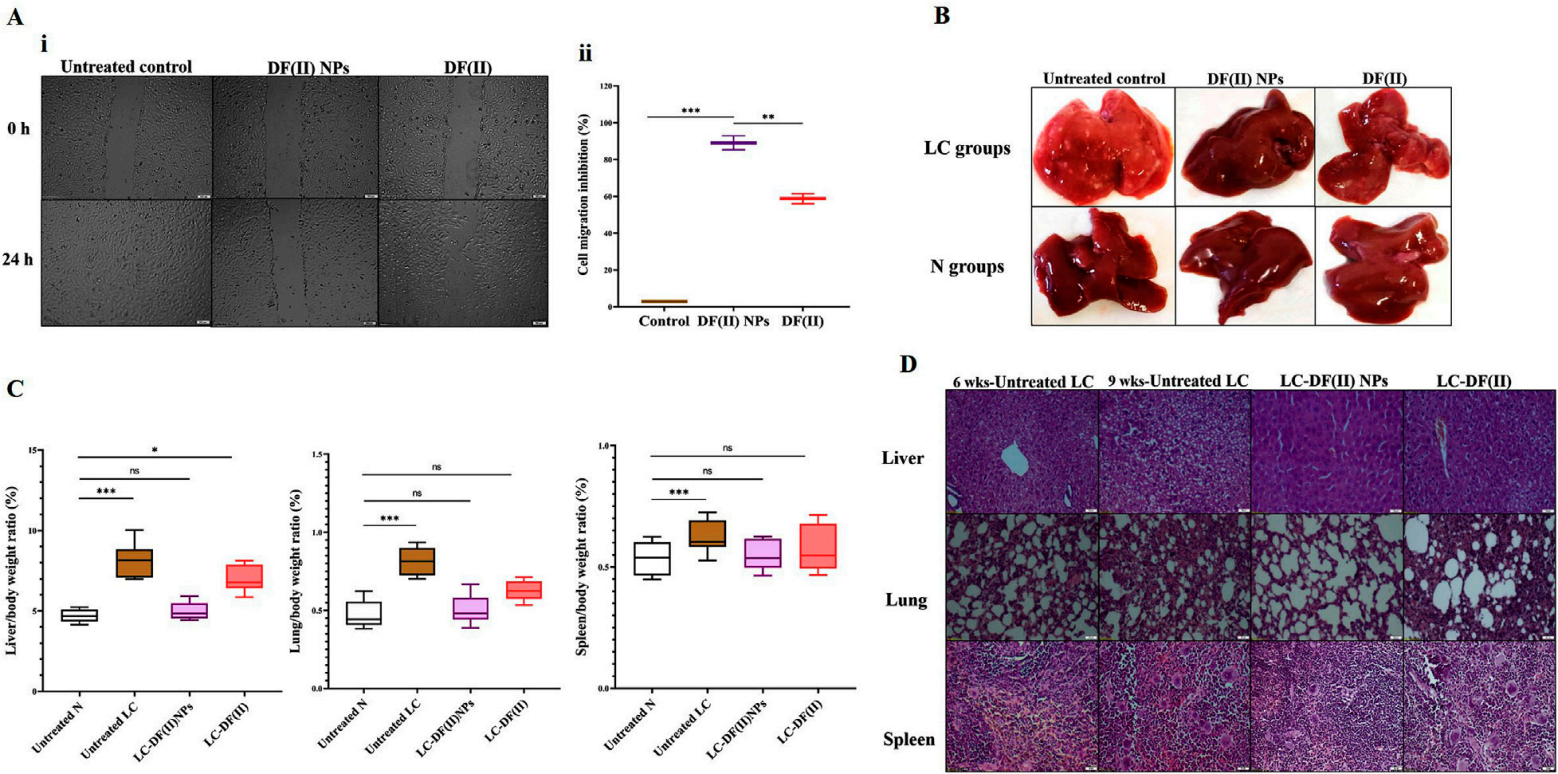
*In vitro* anti-migration potential and *in vivo* anti-liver cancer efficacy (morphology, weight, and histochemical assessments) using chemically induced animal model **(A)** Anti-migration activity as illustrated by **(I)** microscopic images of migration (at 0 and 24 h) of the untreated and DF(II) complexes-treated HepG2 cells and **(II)** migration inhibition percentages. Data are shown as mean ± SEM. DF(II) NPs-treated cells were compared to the untreated and DF(II) complexes-treated cells, considering statistically significant at *p* ≤ 0.05*, ≤0.005**, and ≤0.001***. **(B)** Macroscopic images of liver morphology of all studied groups **(C)** Relative liver, lung, and spleen weights to body weight in the untreated and DF(II) complexes-treated liver cancer (LC) groups, compared to healthy (N) group. Data are shown as mean ± SEM. Healthy N group was compared to the untreated and DF(II) complexes-treated LC groups, considering statistically significant at *p* ≤ 0.05*, ≤0.005**, and ≤0.001*** **(C)** H&E-staining liver, lung, and spleen tissues of the untreated LC group, at the 6th week of LC induction (showing neoplasia in liver, small section of lung cancer cells without modifications in spleen) and at the 9th of LC induction (declaring a characterized anaplasia (clear cell hepatocellular carcinoma, expanded area of lung cancer, and lymphoid hyperplasia in spleen), and DF(II) complexes-treated LC groups (3 weeks of treatment), disclosing stronger therapeutic efficacy of DF(II) NPs than DF for eradicating LC cells with halting metastasis to lung or spleen.

For more clarification, flow cytometry analysis for annexin V/PI-stained HepG2 cells was performed, annexin V-stained cells and dual annexin V/PI-stained cells referring to apoptotic population (Q4) and late cell death population (Q2), respectively. The latter population had the highest percentage in both DF complexes-treated cells (>42%) compared to DE, F(II)NPs, and F(II)-treated cells (<15%). Figure 1G (i,ii) also depicted that DF complexes-treated cells had low apoptotic cell populations (≤20%) as well as the percentage of dual stained-late stage cell death population was significantly higher in DF(II) NPs-treated cells (63.42 ± 2.05%) than DF(II)-treated cells (45.45 ± 0.07%). The recorded annexin V-stained populations in two DF(II) complexes-treated HepG2 cells were primarily related to apoptotic potential of DE (Ishak et al., 2014; Abu-Serie, 2018). Meanwhile, the high percentages of dual annexin V/PI-stained population in DF(II) complexes-treated cells (Figure 1G) are mainly due to ferroptotic effect-dependent lipid peroxidation on membrane, which results in membrane integrity loss (Bekric et al., 2022), subsequently dual dyes stained these cells. In terms of migration inhibitory potential, DF(II) NPs revealed stronger activity than DF(II) *via* halting HepG2 wound closure by 89.02 ± 3.84% and 58.75 ± 2.70% in DF(II) NPs- and DF(II)-treated cells (Figure 2A i,ii). The superior anticancer activity of DF(II) NPs on DF(II) is attributed to their nanocharacters (nanosize and semi-spherical shape), which are responsible for enhanced cellular uptake and accumulation in cancer cells (Brandelli, 2020; Sabourian et al., 2020; Zein et al., 2020). It is also linked to cancer overexpressing proteins-stimulated endocytosis, which mediates high NPs internalization (Ho et al., 2008; Huang et al., 2012).

### 3.3 Anti-liver cancer potential of DF(II) NPs surpassing DF(II), in chemically induced metastatic LC animal model, in terms of the following indicators

#### 3.3.1 Morphology, weight, and histology of tumor tissues

Liver organ of the untreated LC group showed oval pale yellow nodules with increasing its relative weight by 1.7 folds as well as lung weight by 1.6 folds without any increase in spleen weight, compared to tissues of healthy N group (Figures 2B,C). The incidence of metastatic LC was assured by histological investigation at the 6th week of chemical induction, using H&E staining that revealed severe neoplastic alterations (multinucleated giant nuclei with increasing nuclear to cytoplasm ratio) in liver, pneumocytic hyperplasia in lung, and no abnormal variations in spleen section (Figure 2D). At the 9th week, H&E staining demonstrated clear cell hepatocellular carcinoma with hyperchromatic pleomorphic nuclei anaplastic cells in liver section, expanding adenocarcinoma lesion in lung tissue, and lymphoid nodular hyperplasia (abnormal expansion of white pulp) in spleen (Figure 2D). In LC-DF(II) NPs group, there were no nodules in liver and no weight gain in any organs (liver, lung, and spleen) compared to N groups. Meanwhile, liver of LC-DF(II) group had small nodules (mean number 8, compared to 16 nodules in the untreated LC) with a significant increase in liver weight compared to N group but no abnormal increase in lung or spleen weight (Figures 2B,C). H&E staining declared that DF(II) NPs had a higher therapeutic efficacy than DF(II) as evidence by complete area of normal liver, lung, and spleen cells in LC-DF(II) NPs, which is contrary to the presence of neoplastic area in liver and lung sections with expansion of splenic lymphatic nodules in LC-DF(II). This confirms DF(II) NPs completely inhibited LC growth and metastasis to lung or spleen (Figures 2B,C).

#### 3.3.2 Immunohistochemical and AFP assessment as well as biodistribution in tumor tissues

**FIGURE 3:**
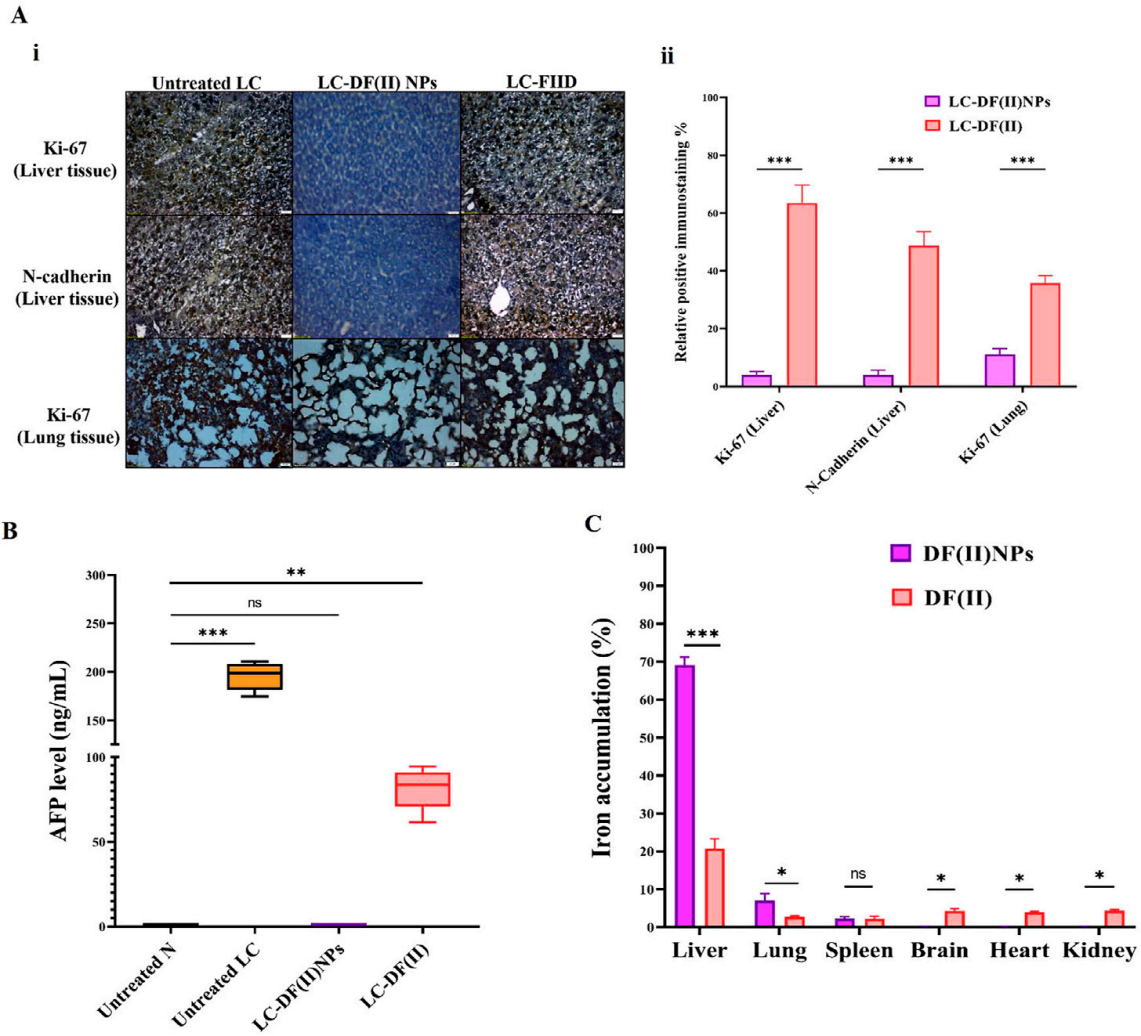
Immunohistological and biochemical investigations of DF(II) complexes impact on tumor markers as well as biodistribution. **(A)** Immunostaining for Ki-67 and N-cadherin in tumor tissues (liver and lung) as illustrated by **(I)** microscopic captured images and (**II**) the percentages of positive immunostained cells. Data are shown as mean ± SEM. LC-DF(II) NPs group was compared to LC-DF(II) group, considering statistically significant at *p* ≤ 0.05*, ≤0.005**, and ≤0.001***. **(B)** Blood level of α-fetoprotein (AFP, ng/mL). Healthy N group was compared to the untreated and DF(II) complexes-treated LC groups, considering statistically significant at *p* ≤ 0.05*, ≤0.005**, and ≤0.001***. **(C)** Biodistribution of DF(II) NPs and DF(II) in liver, lung, and other tissues of the treated LC groups. Data are shown as mean ± SEM. LC-DF(II) NPs group was compared to LC-DF(II) groups, considering statistically significant at *p* ≤ 0.05*, ≤0.005**, and ≤0.001***.

Immunohistochemical detection of ki-67^+^ in tumor tissues (liver and lung) and N-cadherin^+^ (metastasis marker) in liver (Figure 3A i,ii) suggested that DF(II) NPs had significantly stronger suppressive potential for these parameters (95.67%, 88.86%, and 96.02%, respectively) than DF(II) complex (36.45%, 64.22%, and 51.19%, respectively). Furthermore, DF(II) NPs normalized the elevation of AFP which was 325 folds in the untreated LC group compared to N group and moderately decreased to 136 folds in LC-DF(II) group (Figure 3B).

Figure 3C demonstrates that DF(II) NPs accumulated mainly in liver tissue (∼70%) followed by lung (7.0%) and spleen (2.32%), while other tissues of LC-DF(II) NPs group recorded very small amounts (≤0.2%) of this nanocomplex, indicating for a highly selective distribution in tumor tissues. More importantly, hepatic and lung uptakes of DF(II) NPs were >3 folds and 2.6 folds, respectively, higher than those of DF(II) as illustrated in Figure 3C. Based on the enhanced permeability and retention of nanoparticles in tumors (Wu, 2021), the results of atomic absorption declared a selective and massive overload of DF(II) NPs, compared to DF(II), in tumor tissues. This accumulation is the main contributor for inducing targeted and efficient ferroptosis (Bekric et al., 2022). The strong trigger of ferroptosis in LC-DF(II) group interprets the perfect eradication of hepatoma cells (Figures 2B,D), which was accompanied by a halt in the elevation of key tumor protein markers, including Ki-67, N-cadherin, and AFP (Figures 3A,B). The marked upregulation of these mentioned markers indicates a high grade of tumor stage, metastatic growth and poor survival (Sell, 2008; Shi et al., 2015; Kaszak et al., 2020). Therefore, it is important to monitor them.

#### 3.3.3 Redox markers

**FIGURE 4:**
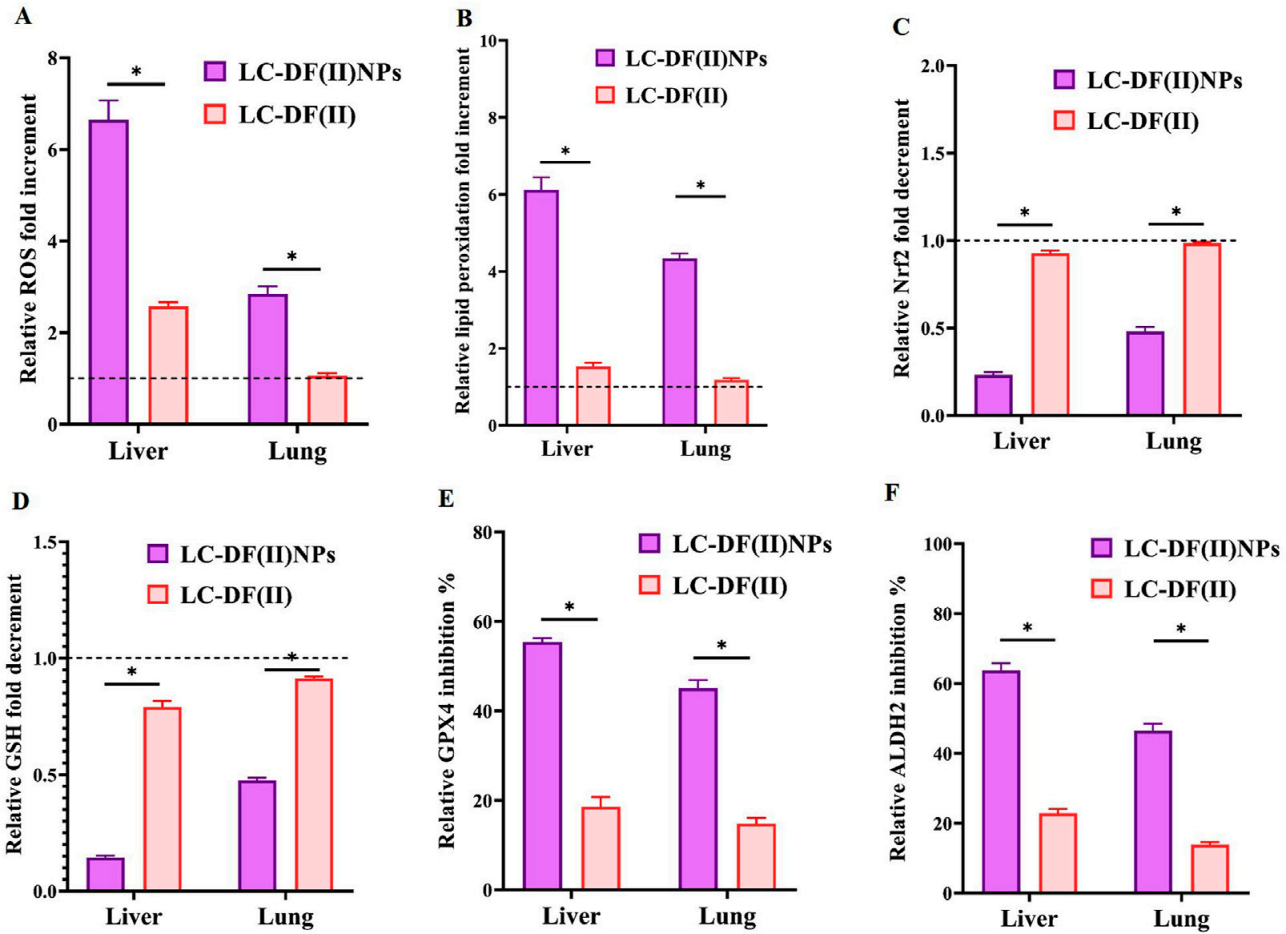
Lipid peroxidation generation-dependent pro-oxidant activity of DF(II) complexes in tumor tissues relative to the untreated LC groups. Relative fold increment in **(A)** reactive oxygen species (ROS) and **(B)** lipid peroxidation. Relative fold decrement in **(C)** Nrf2 transcriptional activation and **(D)** glutathione (GSH), All treated group values were normalized to the untreated LC group values, which were set at 1 as demonstrated by the dashed line, with values above 1 indicating fold increment and values below 1 demonstrating fold decrement. Relative inhibition percentages of lipid peroxide-detoxifying enzymes, including **(E)** glutathione peroxidase (GPX)4 and **(F)** aldehyde dehydrogenase (ALDH) 2. Data are shown as mean ± SEM. LC-DF(II) NPs group was compared to LC-DF(II) groups, considering statistically significant at *p* ≤ 0.05*, ≤0.005**, and ≤0.001***.

These potent anti-LC activities of DF(II) nanocomplex are mainly attributed to its lipid peroxidation generation-dependent pro-oxidant activity as indicated by elevation of ROS and lipid peroxidation in tumor tissues (liver and lung) by 2.8–6.6 folds and 4.3–6.1 folds, respectively, relative to the untreated LC group (Figures 4A,B). Meanwhile, DF(II) increased ROS and lipid peroxidation contents by only 1.06–2.6 folds and 1.18–1.53 folds, respectively (Figures 4A,B). Moreover, DF(II) NPs suppressed Nrf2 transcriptional activity (2.09–4.3 folds), decreased GSH level (2.10–6.94 folds), and inhibited GPX4 and ALDH2 activities (by > 45.02% and >46.50%) more effectively than DF(II) complex (1.06–2.58 folds, 1.18–1.53 folds, ∼1 fold, 1.096–1.26 folds, 2.97–3.04 folds, and 2.78–3.35 folds, respectively) as shown in Figure 4 C-F. These anti-ferroptotic parameters (Nrf2, GSH, GPX4, and ALDH2) were inactivated by the thiol-affinity of DE forming disulfides resulting in preventing the transcriptional activity (promotor binding) of Nrf2, irreversible oxidation of GSH, and inhibiting GPX4 and ALDH activities (Brar et al., 2004; Abu-Serie & Eltarahony, 2021; Nie et al., 2022). These latter enzymes play key roles in the detoxification of lipid peroxide aldehydes (Bekric et al., 2022; Gao et al., 2022). Recent studies have illustrated that ALDH2 protects against these reactive aldehydes (malondialdehyde and 4-hydroxy-2-nonenal) by converting them into malonic acid and hydroxy-2-nonenoic acid, respectively (Zhao et al., 2019; Gao et al., 2022). Due to higher biodistribution of DF(II) NPs in tumor tissues, this antioxidant protective system was lower in LC-DF(II) NPs and subsequently, this group showed stronger elevation of ROS and lipid peroxidation than in LC-DF(II) group.

#### 3.3.4 Molecular tumor markers

**FIGURE 5:**
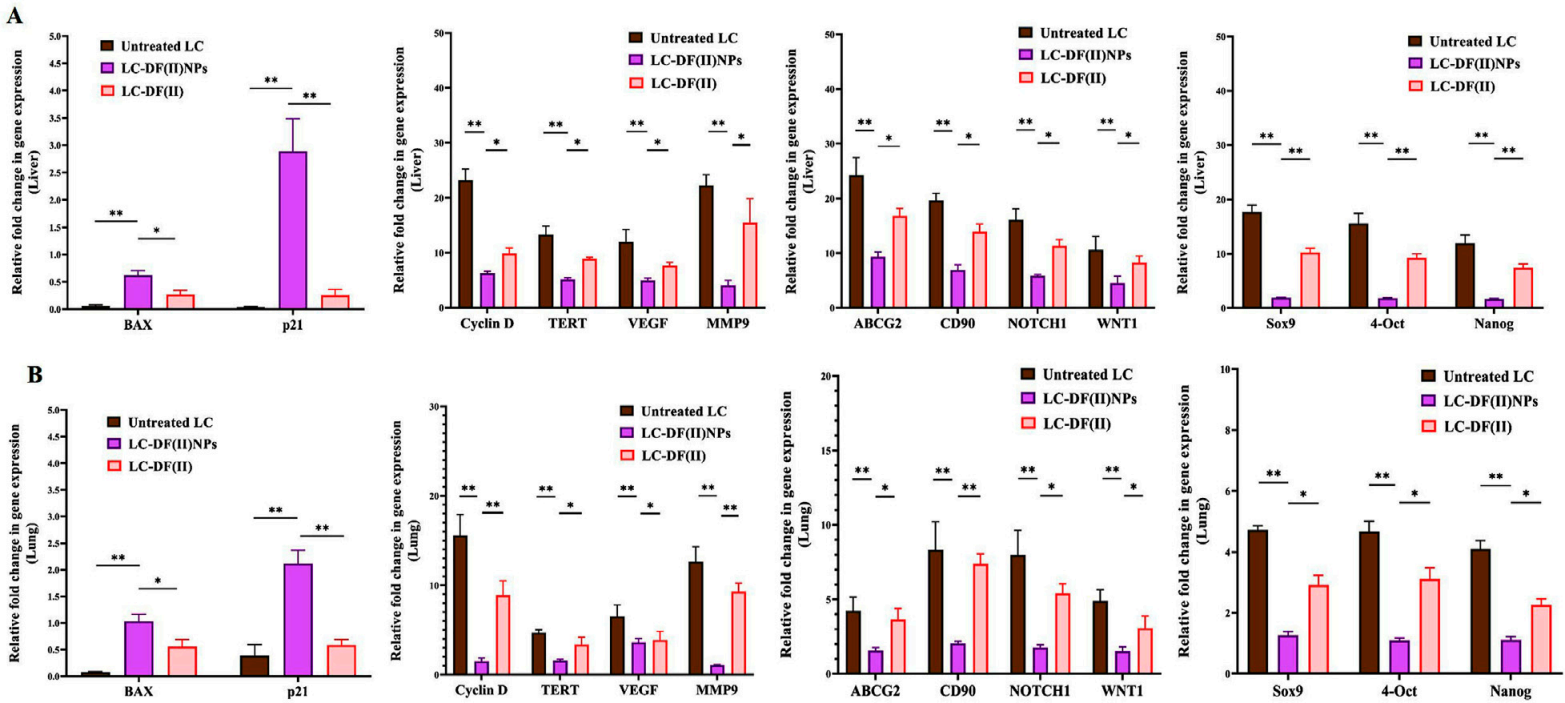
Impact of DF(II) complexes on the gene expression of apoptotic, oncogenes, and stemness genes. Relative fold change in the gene expression of apoptotic genes (BAX and p21), key oncogenes (cyclin D, telomerase reverse transcriptase (TERT), vascular endothelial growth factor (VEGF), matrix metalloprotease (MMP) 9-mediated metastasis), and stemness (ATP Binding Cassette Subfamily G Member 2 (ABCG2), CD90, NOTCH1, WNT1, Sox9, 4-OCT, and Nanog) in tumor tissues, including **(A)** liver and **(B)** lung. Data are shown as mean ± SEM. LC-DF(II) NPs group was compared to the untreated LC and LC-DF(II) groups, considering statistically significant at *p* ≤ 0.05*, ≤0.005**, and ≤0.001***.

Moreover, DF(II) nanocomplex was more effective than DF(II) in upregulating the expression of proapoptotic genes (BAX and p21) and downregulating the expression of all studied oncogenes and stemness genes in tumor tissues of liver and lung (Figure 5 A,B). In both tumor tissues, DF(II) NPs enhanced apoptotic gene expression by 5.5–72 folds and lowered the expression of cyclin D, TERT, VEGF, and MMP9 by ≥ 2 folds and stemness genes (ABCG2, CD90, NOTCH1, WNT1, Sox9, 4-Oct, and Nanog) by ≥ 2 folds compared to <7 folds, ≤2 folds, and ≤1 fold in the case of DF(II), respectively (Figures 5A,B). A previous study illustrated that bismuth diethyldithiocarbamate enhanced cell cycle arrest and apoptosis in HepG2 cells by activation of p53 and BAX (Ishak et al., 2014). This effective alteration in the gene expression of apoptosis, cell cycle, telomerase, angiogenesis, metastasis, chemoresistance, and stemness (NOTCH1, WNT1, Sox9, 4-Oct, and Nanog) in LC-DF(II) is strongly linked to the high accumulative DF(II)-mediated potent ferroptosis-dependent lipid peroxidation, compared to DF(II). As it was demonstrated, DF(II) NPs can suppress functional parameters (ALDH2, CD90, and stemness genes) of CSCs which are responsible for tumor metastasis and chemoresistance (Xiao et al., 2016; Zhang et al., 2018; Wang et al., 2020).

#### 3.3.5 Liver function and hematological parameters

**FIGURE 6:**
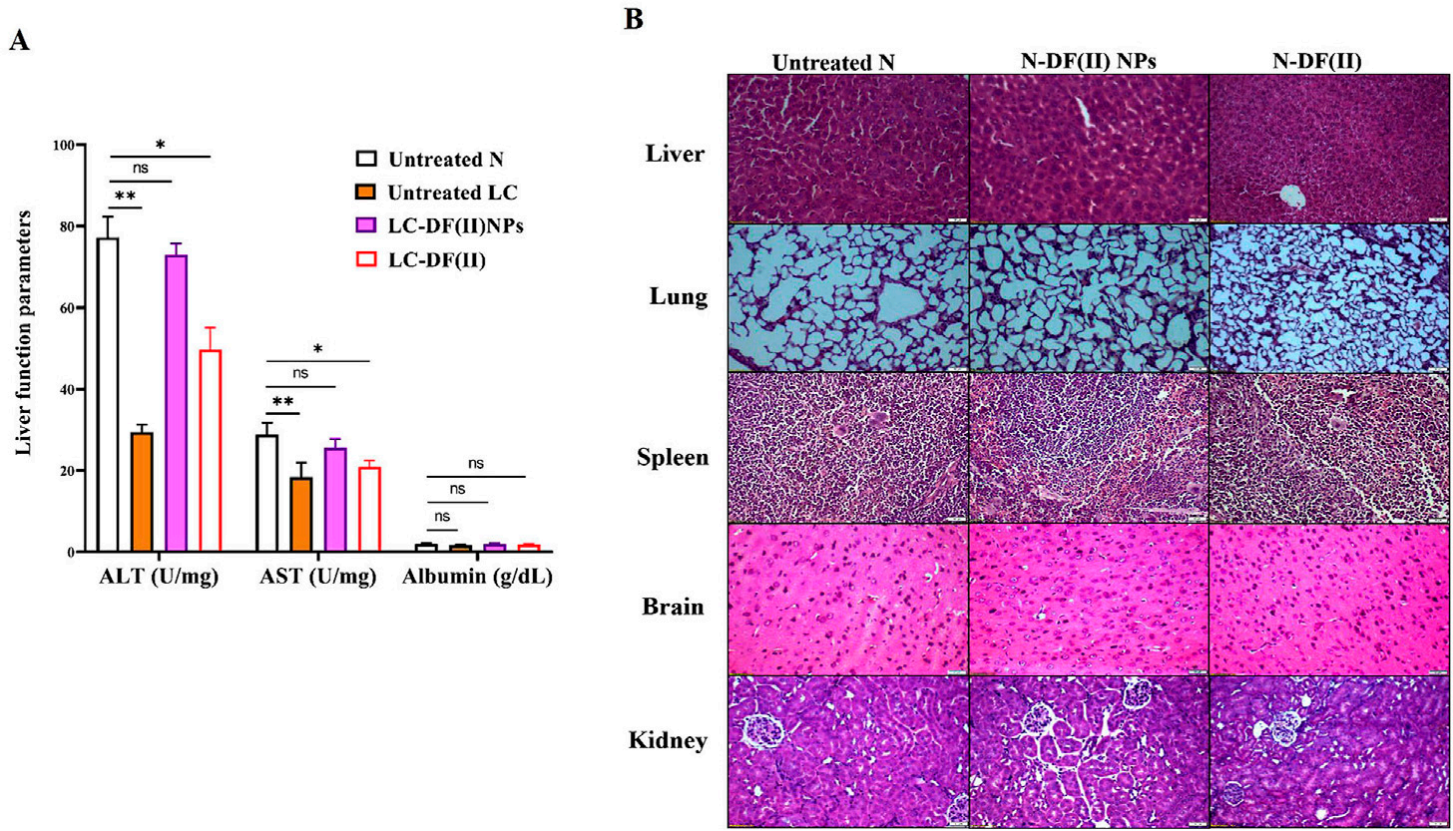
Impact of DF(II) complexes on liver function parameters as well as histological assessment of their toxicity on tissues of the treated N groups **(A)** Liver function indexes (ALT, AST, and albumin) in healthy N group and the untreated and treated LC groups. Healthy N group was compared to the untreated and DF(II) complexes-treated LC groups, considering statistically significant at *p* ≤ 0.05*, ≤0.005**, and ≤0.001*** **(B)** H&E staining tissues of DF(II) NPs- and DF(II)-treated N groups compared to the untreated healthy N groups.

Regarding liver function indexes (Figure 6A), ALT and AST activities were decreased significantly in liver tissues of the untreated LC group (29.49 U/mg protein and 18.37 U/mg protein, respectively) compared to healthy N group (77.26 U/mg protein and 28.82 U/mg protein, respectively). DF(II) NPs were found to be able to normalize this depletion in liver enzymes (72.96 U/mg protein and 25.69 U/mg protein, respectively). On the other hand, DF(II) improved their activities (49.74 U/mg protein and 20.94 U/mg protein, respectively) but still significantly lowered when compared to N group. There was no abnormal variation in the albumin level between the untreated or treated LC groups and healthy N group (Figure 6A). This insignificant difference between the untreated LC group and N group, in the term of albumin level, could be attribute to the protective role of albumin against oxidative damage by preserving cellular GSH (Carvalho & Verdelho, 2018). A recent study declared an inverse correlation between the risk of different cancer types (including, LC) and albumin level (Yang et al., 2021). The normalization effect of DF(II) NPs on the altered liver enzymes ascertained efficient eradication of hepatoma cells.

In terms of hematological indicators (Table 1), the untreated LC groups showed a significant decrease in lymphocyte (Lymph) percentage with increasing monocyte (Mid) and granulocyte (Gran) percentages and no abnormal changes in other parameters relative to healthy N group. These abnormal variations were only normalized in LC-DF(II) NPs group compared to N group and there was no significant improvement in these leukocyte parameters in LC-DF(II) group (Table 1). Lymphopenia is reported in metastatic tumors, particularly non-Hodgkin’s lymphoma, LC, and pancreatic cancer, which suppress the immune response by a variety of mechanisms, including the expression of proapoptotic ligands (Ménétrier-Caux et al., 2019). Therefore, the anti-tumor-dependent ferroptotic effect of DF(II) NPs halted the immunosuppressive potential of metastatic hepatoma cells.

**TABLE 1:** Hematological (WBCs, platelets, and RBCs) parameters.

	WBCs				PLTs			
	WBC (10^3^/µl)	Lymph%	Mid%	Gran%	PLTs (10^3^/µl)	MPV (fl)	PDW	PCT (ml/L)
Untreated N	8.67 ± 0.4	85.8 ± 1.0	5.62 ± 0.6	8.59 ± 0.4	1,211 ± 20	5.99 ± 0.1	15.1 ± 0.2	6.75 ± 0.3
N-DF(II)NPs	8.52 ± 0.6	86.2 ± 1.5	5.35 ± 0.9	8.49 ± 0.5	1,208 ± 26	6.10 ± 0.2	15.2 ± 0.1	6.68 ± 0.2
N-DF(II)	7.11 ± 0.8	81.9 ± 0.3	6.69 ± 0.6	11.4 ± 0.8	1,231 ± 36	5.93 ± 0.1	14.8 ± 0.2	7.37 ± 0.4
Untreated LC	8.64 ± 0.7	63.8 ± 2.2*	14.8 ± 3.5*	21.4 ± 1.3*	1,179 ± 26	5.72 ± 0.2	14.9 ± 0.3	6.36 ± 0.4
LC-DF(II)NPs	8.71 ± 0.3	80.8 ± 2.3	4.29 ± 0.14	14.9 ± 2.5	1,201 ± 6	5.35 ± 0.1	15.0 ± 0.1	7.08 ± 0.4
LC-DF(II)	7.85 ± 0.8	68.8 ± 1.2*	13.1 ± 1.5*	18.1 ± 0.2*	1,184 ± 29	5.60 ± 0.2	15.1 ± 0.1	8.00 ± 0.3

** **	RBCs							
** **	RBC (10^6^/µl)	Hg (g/dl)	HCT (%)	MCV (fL)	MCH (pg)	MCHC (g/dl)	RDW-CV (%)	RDW-SD (fL)
Untreated N	7.34 ± 0.5	12.5 ± 0.5	37.3 ± 2.2	54.1 ± 1.0	17.77 ± 0.3	32.08 ± 0.9	16.8 ± 0.2	29.3 ± 0.6
FND NPs	7.26 ± 0.5	12.7 ± 1.0	38.2 ± 4.0	56.9 ± 2.5	18.10 ± 0.5	31.80 ± 0.6	17.8 ± 0.3	29.7 ± 0.8
N-DF(II)	7.14 ± 0.4	11.9 ± 0.0	37.5 ± 0.2	56.3 ± 0.1	17.65 ± 0.1	31.30 ± 0.2	18.2 ± 0.2	31.8 ± 0.8
Untreated LC	7.08 ± 0.3	11.2 ± 0.0	36.3 ± 3.7	50.4 ± 0.7	16.47 ± 0.4	33.75 ± 0.7	16.2 ± 0.3	29.7 ± 1.3
LC-DF(II)NPs	7.45 ± 0.3	13.2 ± 0.3	36.7 ± 2.1	51.2 ± 1.1	17.75 ± 0.2	35.10 ± 1.3	14.7 ± 0.1	27.5 ± 0.7
LC-DF(II)	7.06 ± 0.4	11.2 ± 0.0	36.2 ± 5.1	50.9 ± 4.1	17.30 ± 0.5	34.05 ± 1.8	16.9 ± 0.4	31.3 ± 3.4

All values are demonstrated as mean ± SEM., All studies groups were compared to untreated N group and the differences were considered significant at *p* ≤ 0.05*, ≤0.005**, and ≤0.001***. WBCs; white blood cells, Lymph; lymphocyte, Mid; monocytes, eosinophils, and basophils, Gran; granulocyte, PLTs; platelets, MPV; mean PLT, volume, PDW; PLT, distribution width, and PCT; plateletcrit. RBCs; red blood cells, Hg; hemoglobin, HCT; hematocrit, MCV; mean corpuscular volume, fL; 10^−15^ liter, MCH; mean corpuscular Hg, MCHC; mean corpuscular Hg concentration, RDW-SD, and RDW-CV; RBC, distribution width-coefficient of standard deviation and variation.

### 3.4 Safety of DF(II) complexes in the treated N groups

When compared to healthy N group, DF(II) NPs-treated N group showed normal liver morphology, whereas N-DF(II)-treated group demonstrated liver damage (Figure 6B). The latter is directly attributable to the unselective DF(II) biodistribution, which resulted in unspecific ferroptosis-mediated undesirable side effects that injured healthy cells (Jia et al., 2021). Other tissues (lung, spleen, brain, and kidney) showed no histological variations (Figure 6B), and normal hematological measurements (Table 1) were normal in both DF(II) complexes-treated N groups (N-DF(II) NPs and N-DF(II)).

## 4 Conclusion

Based on the nanoformulation privilege, the prepared DF(II) NPs exhibited superior therapeutic potential compared to DF(II), against metastatic LC, *via* mediating a selective accumulated iron-dependent ferroptotic effect associated with severe depletion in antioxidant (anti-ferroptosis) response in tumor tissues (liver and lung). Consequently, metastatic tumor mediators (ki-67, N-cadherin, AFP, and gene expression of cyclin D, TERT, VEGF, and MMP9) as well as functional parameters of metastatic seeds (CSCs) were dramatically downregulated in LC-DF(II) NPs. Hence, this nanocomplex can efficiently eradicate hepatoma cells and halt their metastasis to lung, as evidence by histological and immunohistochemical findings. Moreover, DF(II) NPs did not show any alterations in hematological and histological parameters when were administered to normal healthy mice. All these findings support the safety and efficacy of DF(II) NPs in the treatment of metastatic LC.

## Data Availability

The original contributions presented in the study are included in the article/Supplementary Material, further inquiries can be directed to the corresponding author.
