# Erratum: Targeted ferroptotic potency of ferrous oxide nanoparticles-diethyldithiocarba mate nanocomplex on the metastatic liver cancer

**DOI:** 10.3389/fphar.2023.1235710

**Published:** 2023-06-13

**Authors:** Frontiers Production Office

**Affiliations:** Frontiers Media SA, Lausanne, Switzerland

**Keywords:** metastatic liver cancer, selective ferroptosis, ferrous oxide NPs-diethyldithiocarbamate nanocomplex, blocking antioxidant (anti-ferroptotic) mediators, cancer stem cell genes

Due to a production error, there were typing errors made in the article as published.

A correction has been made to the section **Abstract**:

“Thus, F(II) NPs were used with DE as a nanocomplex (DF(II) NPs), whose anti-LC activity was compared to that of the typical complex, DF(II). In HepG2 cells and a chemically induced metastatic LC animal model, DF(II) NPs outperformed DF(II) in eradicating metastatic LC cells, as evidenced by flow cytometry, histological and immunohistochemical analyses, and α-fetoprotein depletion.”

A correction has been made to the section **Introduction**:

“Liver cancer (LC) is a lethal malignancy that affects people all over the world. Because of its rapid proliferation, and migration as well as chemoresistance, its progression is difficult to be controlled with existing treatment options, particularly at the advanced stage (metastasis), resulting in a low survival rate (Li et al., 2022; Zhao et al., 2022). Consequently, finding an alternative remedies becomes a current concern. One of the effective therapeutic strategies is inducing ferroptosis, a new non-apoptotic form of regulated cell death which is characterized by iron accumulation-mediated lipid peroxidation propagation (Bekric et al., 2022; Li et al., 2022). It is important to note that unselective induction of ferroptosis can also damage other healthy hepatic cells and tissues (Bekric et al., 2022). Therefore, a nanoformulation of ferroptotic inducers is critical for improving safety by preventing unspecific side effects and enhancing anti-cancer efficacy.”

The publisher apologizes for this mistake. The original version of this article has been updated.

