# Supplementary material for: Targeted ferroptotic potency of ferrous oxide nanoparticles-diethyldithiocarbamate nanocomplex on the metastatic liver cancer
Source: Front Pharmacol. 2023 Jan 4;13:1089667. doi: 10.3389/fphar.2022.1089667 (PMC9847675; doi:10.3389/fphar.2022.1089667)
Supplement: Supplementary file 1 [file Table1.docx]

**Supplementary Table 1. Forward (F) and reverse (R) primers of the used genes**

| **Gene** | **Primers** |
| --- | --- |
| BAX | \| F: 5′-AGCAAACTGGTGCTCAAGGC-3′ \| \| --- \| \| R: 5′-CCACAAAGATGGTCACTGTC-3′ \| |
| p21 | \| F: 5′-TGCCGCTGCCTCTTTGGT-3′ \|  \| \| --- \| --- \| \| R: 5′-AAAGTCGAAGTTCCATCGCTCT-3′ \| |
| Cyclin D1 | F:5′-CAGAAGTGCGAAGAGGAGGTC-3′  R: 5′-TCATCTTAGAGGCCACGAACAT-3′ |
| TERT | F: 5′-GCACTTTGGTTGCCCAATG-3′  R: 5′-GCACGTTTCTCTCGTTGCG-3′ |
| VEGF | F: 5′-GTGAGGTGTGTATAGATGTGGGG-3′  R: 5′-ACGTCTTGCTGAGGTAACCTG-3′ |
| MMP9 | F: 5′-GCGTCGTGATCCCCACTTAC-3′  R: 5′-CAGGCCGAATAGGAGCGTC-3′ |
| ABCG2 | F: 5′-GAACTCCAGAGCCGTTAGGAC-3′  R: 5′-CAGAATAGCATTAAGGCCAGGTT-3′ |
| CD90 | F: 5′-ACCAAGGATGAGGGCGACTA-3′  R: 5′-ACAGGCACAGTCCAACTTCC-3′ |
| Notch1 | F: 5′-CCCTTGCTCTGCCTAACGC-3′  R: 5′-GGAGTCCTGGCATCGTTGG-3′ |
| Wnt1 | F: 5′-GGTTTCTACTACGTTGCTACTGG-3′  R: 5′-GGAATCCGTCAACAGGTTCGT-3′ |
| Sox9 | F:5′-AGCTCACCAGACCCTGAGAA-3′  R: 5′-TCCCAGCAATCGTTACCTTC-3′ |
| 4-Oct | F: 5′-CCCACTTCACCACACTCTACT-3′  R: 5′-GCTCCAGGTTCTCTTGTCTA-3′ |
| Nanog | F:5′-CCACCAGGTGAAATATGAGAC-3′  R: 5′-GGCTCACAACCATACGTAAC-3′ |
